# New Pathways to Health Equity: How Implementation Science Can Lead Global Learning to Transform US Healthcare

**DOI:** 10.5334/aogh.5047

**Published:** 2026-05-27

**Authors:** Kevin P. Fiori, Alexander Plum

**Affiliations:** 1 Department of Pediatrics, Division of Community and Population Health, Albert Einstein College of Medicine, Bronx, NY, United States; 2 Corner Health Center, Ypsilanti, MI, United States

**Keywords:** global learning, Implementation science, health equity

## Abstract

The US healthcare system, despite its resources, faces persistent health inequities. Paradoxically, highly effective, community-centric innovations often emerge from resource-constrained low- and middle-income countries. This paper describes how the field of global learning can be an approach for translating these global insights into actionable strategies and that implementation science (IS) provides a methodology to guide the process. Two case studies are described as examples: (1) the adaptation of Community Health Worker best practices from Togo to the Bronx, New York, to address local health disparities; (2) the localized, integrated care model at Michigan’s Corner Health Center, which adopted principles from women’s groups in India. By applying an IS framework to global learning innovations, the US can effectively adapt proven global solutions to close domestic health gaps and foster a more resilient healthcare system.

## Introduction

The pursuit of health equity remains a global challenge and requires new tools and approaches considering unprecedented policy shifts. While high-income countries often have access to advanced technologies and substantial financial resources, paradoxically, some of the most profound innovations in healthcare delivery emerge from environments of scarcity. Communities facing resource constraints frequently devise ingenious solutions born out of necessity, resilience, and deep understanding of local needs. Based on our experience, we see an opportunity for the United States healthcare system to display humility and seek out innovation beyond its borders to fix some of its intractable healthcare problems. We propose that the Global Learning Network for Health Equity (GLNHE) framework [1], coupled with rigorous implementation science (IS) methodologies, provides a new pathway for translating these global insights into actionable strategies to address health disparities.

### Innovation born of necessity: lessons from outside our borders

In many low- and middle-income countries (LMICs), healthcare systems operate under immense pressure, characterized by limited infrastructure, insufficient personnel, and constrained budgets. Yet, it is precisely within these challenging environments that innovation, particularly advancements in health equity, has developed [1]. These are usually adaptive, community-centric, and often low-cost solutions that prioritize accessibility, affordability, feasibility, and local relevance [2]. Some examples would include the following: task-shifting models where non-physicians deliver essential services [3], community-based health worker programs that extend care to remote populations [4], frugal adaptations in medical device design [5], and integrated care approaches that address social determinants of health directly within communities [6].

These innovations often share common characteristics, such as being deeply embedded in local social and cultural contexts [7], driven by community participation, and designed to maximize impact with minimal resources [8]. They highlight a fundamental principle: true innovation is not solely a product of abundance, but often a testament to human ingenuity in overcoming constraints and barriers. Learning from contexts where resourcefulness, unencumbered by legal and regulatory barriers, is a daily imperative can offer unique perspectives on delivering high-quality, equitable care [9]. It challenges the prevailing assumption that more resources automatically lead to better outcomes, urging a focus instead on how existing resources are optimized.

### The global learning network for health equity framework

Recognizing the potential for reciprocal learning, a network of diverse health systems, health departments, and community-based organizations was created to formalize an approach for adapting global health equity ideas. Supported by funding from the Robert Wood Johnson Foundation and hosted by the University of Maryland, Baltimore, the network [10] developed a GLNHE framework that offers a structured approach to facilitate this exchange. The framework draws on principles from the field of health innovation transfer between LMICs, at times referred to as “reverse innovation” [11] and “reciprocal innovation” [12]. It also leverages insights from successful US-based health equity-focused global learning projects involving US health systems [13, 14]. While not a unidirectional transfer of “best practices” from high-income to low-income settings, or vice versa, the framework champions reciprocal learning, where insights, challenges, and solutions are shared across contexts, fostering mutual growth and advancement.

At its core, the global learning framework emphasizes:

1. **Equity-focused dialogue:** Prioritizing discussions around systemic inequities and examining how different contexts have attempted to address them.
2. **Context-sensitive adaptation:** Acknowledging that solutions are rarely universally applicable and must be carefully adapted to specific local conditions.
3. **Shared problem-solving:** Building trust across collaborative environments where diverse stakeholders can collectively analyze problems and co-create solutions.
4. **Community-centricity:** Respecting community voices and lived experiences as the driver of identifying needs, designing and adapting interventions, and evaluating impact.

The global learning framework serves as a starting point, providing the scaffolding for cross-contextual learning. However, the act of translating knowledge and adapting interventions across vastly different healthcare landscapes is far from straightforward. It requires more than just humility, goodwill, and shared intent, but rather it requires methodologies and systematic processes to navigate the complexities of optimizing implementation, including adaptation.

### Using implementation science as a tool for optimization

While the global learning framework provides the “what” and “why” of global learning for health equity, IS offers the crucial “how.” IS is the study of methods to promote the systematic uptake of research findings and other evidence-based practices into routine practice, and, consequently, to improve the quality and effectiveness of health services [15]. Over the past two decades, the field has developed a robust theoretical and methodological base, including frameworks, models, and theories designed to understand and address the gap between evidence and practice [16]. Applying these frameworks and models has been particularly consequential in global health, where context, culture, and resource consideration demand careful attention to adaptation rather than simple replication [17].

It is particularly useful for advancing global learning for health equity because it explicitly addresses the challenges of how to adapt based on changing contexts and real-world barriers and facilitators. These key factors are often overlooked in traditional knowledge transfer models.

Some key approaches from IS that may be directly applicable to global learning for health equity include:

- **Localizing evidence-based interventions:** IS emphasizes that an intervention proven effective in one setting cannot simply be “copied and pasted” elsewhere. It must be carefully localized, meaning its core components are preserved while its implementation strategies, contextual framing, and operational details are adapted to fit the new environment.
- **Ensuring fit for diverse settings:** This goes beyond mere translation. It involves a deep dive into the practical realities of a new context, assessing its readiness for change, identifying potential facilitators and barriers, and understanding the existing workflow and capacities. IS provides tools and frameworks, such as the Consolidated Framework for Implementation Research [18] to systematically identify, summarize, and analyze these factors and guide the adaptation process.
- **Using iterative, participatory approaches:** Successful implementation is rarely a linear process. It requires continuous learning, feedback loops, and adjustments. Participatory approaches, where implementers, beneficiaries, and other key stakeholders are actively involved in the design, testing, and refinement of interventions, are central. This co-creation ensures that solutions are not only evidence-based but also relevant, acceptable, and sustainable within the target community. IS provides key frameworks such as the Dynamic Adaptation Process that provide guidance for this iterative approach [19]. Our experience over the past two decades related to global learning, spanning various health challenges and geographical locations, demonstrates support for these principles. IS provides the methodological toolkit to translate lessons across borders, ensuring that adaptations were deliberate and informed, ultimately helping to close health gaps and scale what works, where and for whom it matters most.

### Case study 1: translating community health worker best practices—togo to the bronx

One illustration of global learning for health equity, guided by IS, is the adaptation of Community Health Worker (CHW) best practices from Togo to the Bronx, New York. In Togo, CHWs are foundational to the healthcare system, often serving as the primary link between remote communities and formal health services. Their model is characterized by deep community integration, task-shifting for essential health services (e.g., basic maternal and child health, acute care case finding), and robust, albeit resource-constrained, systems for supervision and support. These CHWs are highly trusted, culturally competent, and adept at navigating local social networks to deliver care effectively and efficiently. This CHW model has been shown in two studies to significantly reduce child mortality in Togo, with a five-year longitudinal evaluation suggesting a decline in under-5 mortality from 51.1 to 35.8 per 1000 live births with doubled rates of home-based CHW treatment [20], and a subsequent cluster randomized trial across four districts confirmed a 29% reduction in under-5 mortality at scale [21].

Bronx County, New York, despite being in a high-income country, faces significant health disparities, including high rates of chronic disease, maternal mortality, and limited access to primary care, particularly in underserved neighborhoods [22]. Recognizing the potential of CHW models to bridge these gaps, the CHW Institute in the Bronx embarked on a journey of global learning. Rather than simply importing the Togolese model, the institute, guided by IS principles, focused on adapting the core tenets of effective CHW programs [23].

Key adaptations included:

- **Professional development:** Togolese CHWs often receive practical, on-the-job training; the Bronx initiative emphasized formal professional development, certification, and career pathways to integrate CHWs more fully into the health system’s workforce.
- **Supervision and coaching:** Learning from Togo’s structured, supervisory networks, the Bronx program developed formalized systems for ongoing supervision and coaching. This included regular one-on-one sessions, peer learning groups, and continuous professional development tailored to the complex needs of the Bronx workforce.
- **Addressing social needs:** The Togolese model inherently addresses social determinants due to its community embeddedness. In the Bronx, this translated into explicit training for CHWs to serve as a bridge between health and social sectors by connecting patients with resources for housing, food, employment, and legal aid.

This cross-contextual learning, facilitated by an IS lens, allowed the Bronx CHW Institute to model best practices in supervision, coaching, and professional development. It demonstrated how a core intervention (CHW-led care) could be adapted to a vastly different resource context, leveraging global insights to address local health equity challenges effectively [23].

### Case study 2: adapting women’s groups for maternal infant healthcare—from india to michigan’s corner health center

Another powerful example, though perhaps more focused on localized innovation that offers lessons for global learning, is the work of the Corner Health Center in Ypsilanti, Michigan. As the state’s first and largest medical center devoted to the care of young people aged 12–25, the Corner’s innovative approach to youth-centered, integrated healthcare services fundamentally rethinks how healthcare is delivered to a population often marginalized and underserved by traditional systems.

Leaders from the Corner’s maternal-infant health program (MIHP) partnered with an NGO based in Himachal Pradesh, India, to learn lessons from their success animating rural women’s empowerment groups. Those lessons are crystallized as the NGO’s “4 Pillars” approach to women’s empowerment: participation, integration, networking, and sustainability.

Because the Corner shared similar core values prioritizing these “4 Pillars” among low-income women MIHP patients in their own health journeys, a pilot project was initiated to adapt the NGO’s model of 4 Pillars Women’s Groups among MIHP patients at the Corner. A rigorous adaptation design and evaluation process involving over 100 MIHP patients and Corner staff resulted in the implementation of a new perinatal health program for women called Parent Cafés [24].

The Corner Health Center’s approach highlights several key elements relevant to global learning and IS:

- **Youth-centered design:** MIHP services were adapted *with* and *for* young women, who capitalized on Corner’s welcoming, non-judgmental reputation to advocate for dedicated program time to socialize with their peers while childcare is provided. This mirrors the GLNHE’s emphasis on community voice.
- **Integrated care:** By co-locating medical, mental health, and social services, the Corner’s MIHP addresses the holistic needs of young women, recognizing the special interconnectedness of physical health, mental well-being, and social determinants during the perinatal period. This integrated approach, central to India’s success in resource-constrained settings where efficiency is paramount, offers valuable lessons for fragmented high-income systems.
- **A network of accessibility and affordability:** The Corner works to remove barriers to care, offering services regardless of ability to pay and navigating insurance complexities. This focus on practical accessibility directly follows from its network of nonprofit partners responding to other facets of MIHP patients’ needs. Embodying the oft-cited adage “if you want to go far, go together,” the Corner’s approach is a direct echo of innovations in LMICs that prioritize reaching vulnerable populations by maximizing shared resources.
- **Community embeddedness:** While funding allocations and insurance reimbursement have waxed and waned over its 45 years of operations, the Corner’s deep roots in the Ypsilanti community and its understanding of local youth culture allow it to build trust, effectively engage its target population, and sustain its mission. From an IS perspective, the success of the Corner’s MIHP pilot can be attributed to its iterative adaptation of nonclinical services to meet evolving youth needs, its continuous engagement with its pregnant young clients as key stakeholders in service design, and its persistent efforts to overcome systemic barriers to adolescent health. Its model can serve as a valuable case study for other communities, both within the US and globally, seeking to improve health outcomes for young people. It demonstrates how a deep understanding of a specific population’s context, combined with a commitment to integrated, accessible care, can lead to highly effective and equitable health service delivery. Learning from such localized successes, and systematically understanding *how* they achieve their results, is a critical component of global learning for health equity.

### Challenges and future directions

While the potential of global learning for health equity, guided by IS, is immense, the path to widespread adoption is not without significant challenges. The act of translating and adapting interventions across diverse contexts is inherently complex. The authors’ experiences working in large integrated health systems have borne out this fact, within both community and care delivery environments. Barriers they have experienced include:

- **Policy and regulatory environments:** Differing healthcare policies, licensing requirements, and regulatory frameworks can impede the seamless transfer and adaptation of models from one location to another.
- **Cultural resistance and mindset shifts:** Overcoming ingrained beliefs, professional silos, and resistance to change within established systems requires deliberate effort and strong leadership buy-in.
- **Financing and sustainability:** Despite the proliferation of value-based funding schemes, the US healthcare system still largely relies on fee-for-service billing, which is poorly structured for community-focused, upstream interventions common in global learning. Finding ways to incorporate these elements into funding schemes is essential to sustain their uptake.
- **Expertise:** Most community-based and grassroots organizations lack the expertise, time, or resources to engage in IS processes at a rigorous level. Basic approaches as outlined in this article should be more accessible to wider audiences. This can be aided by more community-centric academic-community partnerships and an effort within the IS field to make its work more practical at a community level.

IS’s gift to the field of global learning is the rigor and systematized methodology it offers to practitioners looking to achieve health equity at home. Yet it is not a panacea. IS cannot rewrite federal policy or change an organization’s innovation culture. What it can do is provide committed change agents with an approach to include contextual realities to overcome these challenges.

## Conclusion

Innovation in healthcare is not exclusive to environments of technological advancement or financial abundance. Indeed, some of the most profound and equitable solutions emerge from communities that, driven by scarcity, develop highly effective, community-centered approaches to health. The US healthcare system has a unique opportunity to learn from these communities and integrate these lessons to address its own deeply entrenched health inequities. The GLNHE framework provides the vision for such reciprocal exchange, but it is IS that offers the vital methodologies to bridge the gap between theory and practice. To that end, the authors offer the following recommendations for strengthening this work:

- **Investment in IS capacity:** Building a cadre of researchers and practitioners skilled in IS methodologies is crucial for systematic adaptation and evaluation.
- **Flexible funding mechanisms:** Developing funding models that support iterative, adaptive implementation processes rather than rigid, pre-defined interventions.
- **Policy advocacy:** Engaging policymakers to create enabling environments that facilitate cross-contextual learning and the adoption of innovative, equity-focused models.
- **Strengthening global learning networks:** Continuously fostering and expanding platforms like the GLNHE to facilitate deeper, more reciprocal partnerships and knowledge exchange.
- **“True” reciprocity:** Ensuring that learning is genuinely bidirectional and that insights from LMICs are valued and integrated into high-income country strategies, rather than merely being seen as “lessons for them,” requires a fundamental shift in perspective.

By emphasizing adaptation, context, and genuine stakeholder engagement, IS transforms abstract global lessons into tangible, localized interventions. As demonstrated by the successful adaptation of CHW models from Togo to the Bronx and the innovative youth-centered care at Corner Health Center, this approach can effectively translate insights across borders, close health gaps, and ultimately foster a more equitable, resilient, and responsive healthcare system for all. Embracing global learning, underpinned by IS, is not just an academic exercise; it is a new pathway for transforming healthcare delivery.
